# Bed Rest, Exercise Countermeasure and Reconditioning Effects on the Human Resting Muscle Tone System

**DOI:** 10.3389/fphys.2018.00810

**Published:** 2018-07-03

**Authors:** Britt Schoenrock, Vanja Zander, Sebastian Dern, Ulrich Limper, Edwin Mulder, Alar Veraksitš, Ragnar Viir, Andreas Kramer, Maria J. Stokes, Michele Salanova, Aleko Peipsi, Dieter Blottner

**Affiliations:** ^1^Vegetative Anatomy, Charité Universitätsmedizin Berlin, Berlin, Germany; ^2^Neuroscience Group, German Sports University, Cologne, Germany; ^3^Human Physiology, German Aerospace Center, Cologne, Germany; ^4^Institute of Biomedicine and Translational Medicine, Faculty of Medicine, University of Tartu, Tartu, Estonia; ^5^Ragnar Viir, Limited Partnership, Helsinki, Finland; ^6^Neuromechanics Research Group, Sport Sciences, University of Konstanz, Konstanz, Germany; ^7^Faculty of Health Sciences, University of Southampton, Southampton, United Kingdom; ^8^Center of Space Medicine Berlin, Berlin, Germany; ^9^Myoton AS, Tallinn, Estonia

**Keywords:** skeletal muscle, tendon, fascia, biomechanical properties, disuse

## Abstract

The human resting muscle tone (HRMT) system provides structural and functional support to skeletal muscle and associated myofascial structures (tendons, fascia) in normal life. Little information is available on changes to the HRMT in bed rest. A set of dynamic oscillation mechanosignals ([Hz], [N/m], log decrement, [ms]) collected and computed by a hand-held digital palpation device (MyotonPRO) were used to study changes in tone and in key biomechanical and viscoelastic properties in global and postural skeletal muscle tendons and fascia from a non-exercise control (CTR) and an exercise (JUMP) group performing reactive jumps on a customized sledge system during a 60 days head-down tilt bed rest (RSL Study 2015–2016). A set of baseline and differential natural oscillation signal patterns were identified as key determinants in resting muscle and myofascial structures from back, thigh, calf, patellar and Achilles tendon, and plantar fascia. The greatest changes were found in thigh and calf muscle and tendon, with little change in the shoulder muscles. Functional tests (one leg jumps, electromyography) showed only trends in relevant leg muscle groups. Increased anti-Collagen-I immunoreactivity found in CTR soleus biopsy cryosections was absent from JUMP. Results allow for a muscle health status definition after chronic disuse in bed rest without and with countermeasure, and following reconditioning. Findings improve our understanding of structural and functional responses of the HRMT to disuse and exercise, may help to guide treatment in various clinical settings (e.g., muscle tone disorders, neuro-rehabilitation), and promote monitoring of muscle health and training status in personalized sport and space medicine.

## Introduction

In physiological conditions, the resting muscle tone constitutes only about 1% of MVC ability (Woledge et al., 2003) but appears to be sufficient for body stabilization whether being reclined, sitting or relaxed standing in quiet gravity-neutral body positions (Gurfinkel et al., 2006; Loram et al., 2007). There is minimal energy costs (7% supine) and often little fatigue over long durations (Masi and Hannon, 2008).

The passive nature of human body stability on Earth is based primarily on what was previously called the autonomous tone and elasticity component of muscle that counteracts normal movement and inertia during gravitational loading and adaptation to life on Earth (Kozlovskaya et al., 1988; Gallasch and Kozlovskaya, 1998). Myofascial elasticity and tone of the so called HRMT system provides postural stability via anti-gravity forces inherent to intrinsic passive structural body elements such as fascia, tendon and skeletal muscle (Masi and Hannon, 2008). These structures are linked to myofascial kinematic chains traversing through the whole human body to support, for example, lumbopelvic stability and static posture during habitual performance (Willard et al., 2012; Wilke et al., 2016) and to allow optimal force transmission adjustment during exercise (Fitts and Widrick, 1996; Findley et al., 2015). The state of tension of a fully relaxed muscle in the HRMT is reflected by the passive tone (inherent tension) of supposedly resting muscle earlier referred to as rhythmically micro-movements or micro-vibration (Rohracher, 1962), or as viscoelastic tone (specific tone), a vital property of striated muscle at low tension resistent to strain but different from contractile activity such as EMG-silent contractures, normal contraction, or even pathological electrogenic spasms (Simons, 2008).

In routine practice, determination of the resting muscle tone helps to clinically differentiate the underlying complex mechanisms causing detectable tension and stiffness reflected by subjective palpation of altered muscle firmness and rigidity (increased versus normal tone), for example found in the context of pathological changes with muscle pain (Simons and Mense, 1998). Also, this altered tone can be palpable during physical examination in athletes by experienced medical doctors and therapists, as symptomatic increased firmness or hardness following muscle injuries in sports (Mueller-Wohlfahrt et al., 2013). However, it is largely unknown if, for example, passive muscle tone and biomechanical properties such as stiffness and elasticity or viscoelastic characteristics such as mechanical stress relaxation time and creep of myofascial structures are either weakened or reinforced by disuse in long-term bed rest, which particular types of muscles (e.g., global vs. postural) are potential targets for disuse-induced changes in tone, biomechanical and viscoelastic property changes. It is also unknown if physical exercise as a countermeasure is able to recover changes to the HRMT following de- and reconditioning of the human movement system.

Bed rest is a chronic inactivity model to study adaptation and atrophy mechanisms in disuse-induced wasting conditions (Bodine, 2013), and to test for efficacy of physical counter-measures to alleviate disuse-induced bone and muscle wasting (Pavy-Le Traon et al., 2007; Hargens and Vico, 2016), as well as neuromuscular performance loss (Mulder et al., 2009; Salanova et al., 2011) in otherwise healthy volunteers. Changes in general skeletal muscle tone, which is supposed to develop consistently over time in the almost relaxed musculature of the partially unloaded human body at supine position during bed rest confinement has been studied in atrophic paraspinal lower back multifidus and lower extremity calf triceps muscle (Koryak, 2014; Holt et al., 2016). These studies suggested a possible link between muscle tonicity changes and the magnitude of bed rest-induced muscle deconditioning characterized by loss in muscle mass and function.

Nevertheless, little information is currently available on tone, biomechanical and viscoelastic property changes (tone/tension, dynamic stiffness, elasticity, stress relaxation time) to the chronically inactive human skeletal muscle and associated myofascial complex that shape the HRMT system following extended disuse in bed rest.

In the present study a hand-held and non-invasive digital palpation device (MyotonPRO) was used to measure changes in myotonometric characteristics and viscoelastic response of individual resting muscle, tendon and fascia of the HRMT in long-term bed rest (60 days RSL Study 2015-2016, DLR:envihab human physiology facility, Cologne, Germany). The measurement method is based on registration of oscillation acceleration signals and subsequent computing of parameters reflecting tone – indicated by oscillation frequency [Hz], dynamic stiffness [N/m], elasticity – indicated by logarithmic decrement [log decrement] and mechanical stress relaxation time [ms]). Myotonometry has proven to provide reliable data sets in previous preclinical feasibility and clinical reliability pilot studies from superficial reference muscles (multifidus, rectus femoris, biceps brachii) in stroke patients (Fröhlich-Zwahlen et al., 2014), Parkinson disease (Marusiak et al., 2012), in normal healthy young and older males and females (Ditroilo et al., 2011; Agyapong-Badu et al., 2013; Nair et al., 2016), as well as in aging (Agyapong-Badu et al., 2016). Recordings using myotonometry were also found to be robust from a calf muscle during short duration weightlessness conditions in the parabolic flight analog (Schneider et al., 2015).

We hypothetized that (i) changes in parameters measured during prolonged bed rest are potential signatures for the known disuse atrophy changes in a region and tissue-specific pattern (global vs. postural), (ii) key myotonometric characteristics of functional muscle groups addressed by a highly demanding countermeasure protocol (reactive jumps on sledge system, RSL) during bed rest could be defined, and (iii) particular biomechanical signal patterns (Hz, N/m, log decrement, ms) were identifiable reflecting HRMT system recovery in participants returning back to normal upright body position and daily activities following post bed rest reconditioning. In addition, muscle physiology data collected from the jumping-related functional muscle chain were used for correlation with biomechanical data for validation and better interpretation of study outcomes. Finally, the intramuscular collagen-1 network was studied in tissue cryosections from RSL SOL biopsies (postural reference muscle) as a potential target of extra- and subcellular tissue remodeling related to differential biomechanical and viscoelastic properties found in the HRMT system in bed rest.

The present study aimed to provide further insights to the structural and functional responses of the HRMT system to chronic disuse during bed rest without (reflected by bed rest effects in a CTR), with exercise as a countermeasure (reflected by reactive jumps performed by an exercise group in bed rest), and following recovery thereafter (reflected by reconditioning effects vs. both groups). The study outcome myotonometry could be used for real time monitoring of musculoskeletal health status in an apparently healthy population, and potentially in spaceflight mission crew.

## Materials and Methods

### General Study Information

Myotonometric measurements were performed in a 60 days 6° head-down-tilt bed rest (HDT) study with reactive jumping in a sledge jump system (developed by Novotec, Medical GmbH, Pforzheim, Germany, on behalf of ESA) as a countermeasure. Reactive jumps in a sledge jump system as a countermeasure for long-term bed rest (RSL) study was run with *n* = 24 male volunteer participants randomly assigned to two bed rest groups, Control (CTR, *n* = 12, aged 28.30 ± 5,76 years, body mass index [BMI] 23.52 ± 2,15) and RSL intervention group (JUMP, *n* = 12, aged 29.90 ± 6,57 years, BMI 23.40 ± 1,67), in two campaigns (1st campaign, August 28 – November 27, 2015; 2nd campaign, January 26 – April 26, 2016) including either of the two bed rest groups. During ambulatory BDC one participant dropped out of the study due to medical reasons not related to the study resulting in a final *n* = 23 study participants. Before the start of bed rest, the JUMP training was prescribed for familiarization of all participants (BDC-10 to BDC-1) followed by either bed rest alone (CTR) or bed rest with reactive jumps (JUMP) during HDT+1 to HDT+60 at 6° HDT supine position. During the 14 days of BDC and the 15 days of reconditioning (R+0 to R+14) after bed rest, all bed rest participants were allowed to freely move within the ward including a multifocal reconditioning protocol (i.e., 6 × 30 min, R+2 to R+14, tailored to each participant’s status, Kramer et al., 2017b) to improve routine muscle health parameters (e.g., stretching, range of motion, strength, speed, coordination). More detailed information on the RSL sledge jump system, training, loading forces, diet prescriptions, and medical and paramedical treatments can be found in Kramer et al. (2017b). The RSL study was sponsored by the ESA and organized by the German Aerospace Center (DLR) in Cologne, Germany. The whole study was conducted in a novel modular facility (DLR:envihab M2 and M3 module) specialized for human life sciences research^1^. The RSL study was approved by the Institutional Review Board (IRB, Ärztekammer Nordrhein #2014105, Germany) according to the 18th World Medical Assembly of Helsinki, Finland, June 1964, amended by the 41st Assembly, Hong Kong, September 1989. All participants gave written informed consent to take part in the work and were free to withdraw at any stage. The study is registered with the German Clinical Trial registry (#DRKS00012946 on September 18, 2017).

### Myotonometry

**FIGURE 1:**
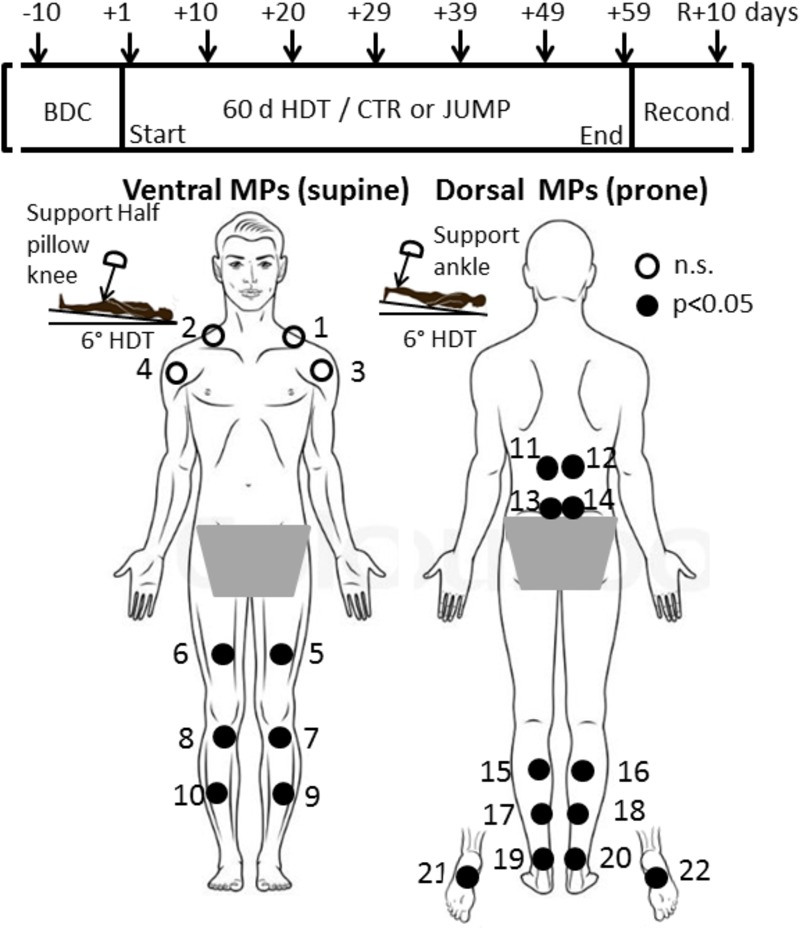
RSL study design and protocol. **(Upper)** Shows time points of myotonometric measurements (arrows) before (BDC), during 6°; head-down tilt bed rest (HDT+1 to HDT+59), and following 10 days of reconditioning (R+10). **(Lower)** With body chart of skin measurement points (MPs) located at upper body, lower back, legs, ankle and foot sole. In a session, myotonometric measurements on premarked skin MPs (closed circles) always started at HDT prone with trapezius MP1/2 (first MP), MP3/4, deltoid; MP5/6, rectus femoris; MP7/8 patellar tendon; MP9/10, tibialis anterior; and supine with MP11/12, erector spine; MP13/14, lumbar multifidus; MP15/16, gastrocnemius; MP17/18, soleus; MP 19/20, Achilles tendon; and plantar fascia/Flexor digitorum brevis, MP21/22 (final MP). A knee and ankle half pillow support was used during supine and prone HDT positioning for leg joint angle standardization in particular (insets).

Myotonometry collected four different biomechanical parameters: Tone [Hz] = F, stiffness [N/m] = S, Elasticity [log decrement) = D, and Relaxation time [ms] = R as computed and shown on the device’s monitor screen (**Figure 1**). Principal outcome interpretation criteria: the higher the values of F and S, the greater the tension and stiffness of the examined soft tissue structure at dedicated body measurement points (MPs). The lower the D value the smaller the dissipation of mechanical energy during oscillation and the higher the elasticity of the muscle (Gavronski et al., 2007), tendon or fascia. The lower the R value the higher the tension or stiffness.

Due to the light and brief mechanical deformation force (pre-load 0.18 N + impulse 0.40 *N* = 0.58 N) over the skin area of 7.01 mm^2^, the minimal mechanical forces produced by the probe on the skin surface do not cause residual mechanical deformation or neurological reactions of the biological tissue of interest being evaluated (**Figure 1**). More details on formulas and outcome measures are found elsewhere (Alev et al., 2018).

### Myotonometry Protocol

Before the start of bed rest, BDC was undertaken. The MPs were identified anatomically on muscle, tendon and fascia structures of interest on each individual bed rest participant, first by manual palpation, followed by marking the skin with crosses using a dermatological skin marker pen. Specific locations of MPs were at the shoulder (upper trapezius: 5 cm distally on virtual line from top of acromion to occipital protuberance; anterior deltoid (*pars clavicularis*): 5 cm distally on a virtual line from acromion to elbow pit at neutral arm position), back (longissimus dorsi: paraspinal Th 12, lateral tract of erector spinae; multifidus: paraspinal L4-5, medial tract of the erector spinae, prone body position), hip (rectus femoris: end of second third distance on a virtual line between anterior superior iliac spine to patella, supine body position), knee (infrapatellar tendon, middle part between patella bottom to tibial tuberosity, supine), leg (gastrocnemius: top of medial head; soleus: distal end of medial gastrocnemius head about 2 cm medially from calf midline; anterior tibialis: 1.5 cm laterally from end of proximal third of the frontal tibial line; Achilles tendon: 3cm distance proximally from medial calcaneus tuberosity, prone position), and sole of the foot (plantar fascia / flexor digitorum brevis: proximal third of virtual line drawn between calcaneus and first and second toe interspace, prone position). The MPs were always re-marked if necessary during the study (when faded away) by either participants or operator. In total, 22 different MPs (2 × 11 from right and left sides) were illustrated in a body chart (**Figure 1**) with dorsal and ventral views (back and frontal view). Full instructions were included in the chart, regarding the appropriate body posture (supine, prone) and exact order of measurements (starting from MP1 to MP22 rostrocaudally in left/right order), to provide additional support to operators to ensure reliable session protocols and data acquisition throughout the study. Before BDC data collection started two operators received supervised training with the digital palpation device (MyotonPRO) for reliable handling and data acquisition from all 22 premarked skin MPs. The sessions always began with the ventral MPs (supine position on bed) followed by the dorsal MPs (prone position on bed). Actual measurements started on the first day of bed rest (HDT+1), then continued in a 10 days time interval (HDT+10, +20, +29, +39, +49) until the end of bed rest (HDT+59), and eventually, after 10 days of ambulatory return from anti-orthostatic (prone, supine) to orthostatic (upright) body position (R+10 reconditioning phase) as shown in **Figure 1** (upper panel).

**FIGURE 2:**
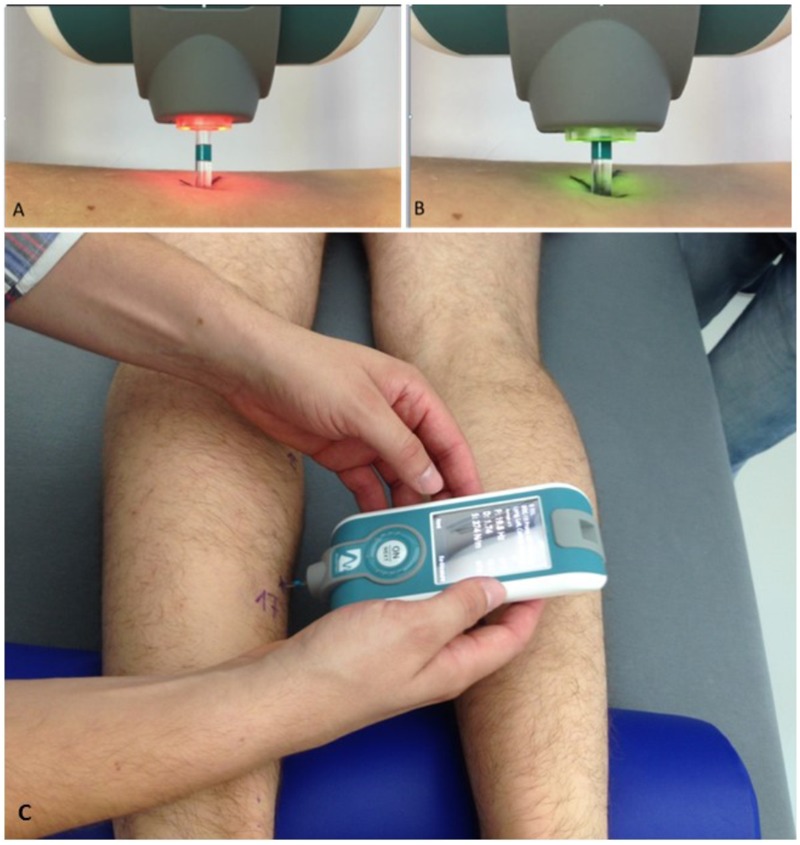
Experimental set-up of myotonometric measurements used in RSL bed rest. Upper panel **(A,B)** shows magnifications of color-coded flat-end probe to show **(A)** inactivated (orange light) and **(B)** activated (green light) sensor probe (vertical tolerance 3 mm) sensor tip. In **(A)**, orange probe shows (re-)positioning phase (0.40N), in **(B)**, green probe shows oscillation acceleration phase ongoing (with x 0.8 s mechanical force intervals). **(C)** Shows the device in perpendicular position with probe gently touching a premarked dorsal leg skin measure point (MP17, soleus) to collect natural oscillation signal patterns in a bed rest study participant lying prone on his own 6° HDT matress at full rest in his study room. Note half-pillow support for standardization of ankle position during sessions. Credit: Myoton AS and D. Blottner.

The general RSL study design with flow chart of time points of myotonometry before, during and after bed rest, and a body chart with skin measure point (MP) locations is presented (**Figure 1**). In **Figure 1**, locations of body MPs with significant changes in biomechanical or viscolelastic properties are depicted as closed circles (open circle MPs denote no change). A magnified image of the digital palpation device sensor head at pre-compression (orange light) and at oscillation acceleration signal state (green light indicating mechanosignal collection) is shown (**Figures 2A,B**). A typical session scenario with myotonometric data collection from calf muscle (soleus) of one RSL study participant (prone position on bed) is illustrated (**Figure 2C**). Briefly, myotonometry involves placing the testing end of the device (**Figure 2**, upper panel), perpendicularly over the premarked skin MP. Gentle compression of the skin surface with the 3mm diameter translucent probe automatically activates a set of minimal mechanical impulses (precompression constant load 0.18N plus impulse 0.40 = 0.58 [N]) i.e., preset series of quick release force at 0.40 Nm followed by five single oscillation acceleration signals at 0.8 ms intervals each. The oscillation signals are instantly computed and stored by the device. In principle, the muscle and myofascial structures (tendon or fascia) located under the subcutaneous compressed tissue respond to the exterior mechanical impulse set by the device itself by damped natural oscillation and co-oscillation signals that are displayed by numerical values on the device’s screen. The oscillation signals are further simultaneously calculated for various given natural oscillation responses [Hz] and other biomechanical and viscoelastic properties [N/m, log decrement, ms]. Because neural activation of the skeletal muscle may likely occur after approximately 25 ms resulting in altered muscle properties, the duration of the probe impact was pre-set at 15 ms to avoid neural reactions and non-elastic deformation signals from the tissue. The low and high frequencies that are not characteristic of natural oscillation patterns of biological soft tissues are filtered out from the raw data sets initiated by the acceleration transducer inside the device. Finally, computed and stored data are transferred from the device via USB to a personal computer for further statistical analysis.

The myotonometric measurements were repeatedly performed according to the body chart instructions (**Figure 1**) within less than a minute for each body MP. The sessions were always performed in a quiet atmosphere in participant’s allocated own study room (M3 module of DLR:envihab facility) after at least 5–10 min of full body relaxation. Participants lay in a natural supine position with arms relaxed by their sides and legs extended, on their own mattress (covered by bed sheet without blanket) on the 6° HDTed beds (prone or supine) to assure conditions of full resting muscle state.

In some cases local MP areas of interest were scanned by ultrasonography to identify proper location and regular tissue architecture of muscle and tendon of interest (Kwah et al., 2013) and to assess potential individual and other potentially hidden tissue alterations (local scarrings from previous injuries/seroma, tiny fluid deposits, edema) in subcutaneous superficial layers that may act as confounders in the soft tissue between probe and muscle and tendon acceleration mechanosignal transmission. Ultrasound scans always followed the myotonometric protocol to avoid potential bias of myotonometric measurements, e.g., unusual skin manipulation, compression and sensation, removing skin gels (data not shown).

### Immunohistochemistry

In the present study, muscle biopsies of the calf soleus (SOL) muscle collected from each participant at three time points (BDC-5, BR+59 and R+10) using the Rongeur forceps technique (Zepf Instrumente, Tuttlingen, Germany) were analyzed. The samples were frozen with liquid nitrogen and stored at -80°C. For immunohistochemistry the samples were cut transversally at 8 μm in a cryostat (CM 1860, LEICA Microsystem) and mounted on coated slides (SuperFrost). In order to quantify the change in extracellular proteins, three single immunostainings were performed on cryocut SOL biopsies only (designated reference postural muscle). We used primary polyclonal antibodies against Collagen I (C-terminus COL1A1, sc-255974), against Collagen I (N-terminus COL21A1 aa 204-232, ab170780). Primary antibodies were diluted 1:50 – 1:100 and incubated overnight at 4°C. Additionally a secondary antibody Alexa 488 (dilution 1:1000, incubation time 1h room temperature) was used. The samples were inspected and images analyzed by a high-signal resolution SP8 confocal microscope^2^ equipped with a hybrid photomultiplier detector and 3 channel multilaser system.

### Countermeasure Exercise Protocol

The JUMP group received a total of 48 training sessions during 60 days of bed rest using a custom-made reactive jump on a sledge (RSL) device (Novotec, Germany). The construction allows a sledge on wheels to move alongside two rails in a 6° HDT frame. During the reactive jump countermeasure session the bed rest participant is fixed to the sledge at supine body position by shoulder straps and able to press their legs during jump movements toward two fixed force plates while the pressing forces generated are counterbalanced by two low pressure cylinders of the device. The sledge position is monitored by an incremental encoder and a feedback monitor which provides live feedback (jump height, peak force) to the participant for each single jump (Kramer et al., 2017b). Randomized group allocation was performed at HDT1, so during BDC there was no distinction between JUMP and CTRL. Before sessions started, study participants were familiarized with the unique training protocol at supine HDT body position prior to all data collection. Due to the highly demanding training protocol the sessions were always preceded by a warm-up period. Countermeasure sessions were prescribed in four different protocol types (A-D) variable in repetitive hops (from 2 × 12 to 4 × 15), amount of countermovement jumps (10 × 3 to 1 × 12), average force loadings (from 80 to 90% of body weight), breaks between series (30 s to 1 min), and training total duration (8–17 min). More detailed information is found elsewhere (Kramer et al., 2017b).

### Jump Tests

Before and after bed rest, jump tests were conducted on a force plate (Leonardo GRFP, Novotec medical GmbH, Pforzheim, Germany) and consisted of one series of 10 one-legged hops, on the left foot with a 1-min break in between. Prior to the first test, all participants were shown and practiced the correct execution of all jumps: hands were placed on the hips and subjects were instructed to jump with maximal effort. The instruction was to “Jump as stiff as possible, i.e., flex the ankle, knee and hip joint as little as possible while still jumping as high as the high stiffness allows; do not let the heels touch the plate during landing, keep the contact time as short as possible and jump as constant as possible.” To be able to trigger the EMG signals the ground reaction forces perpendicular to the force plates were recorded and synchronized to the EMG signals via a data acquisition unit (Power1401-3, CED, Cambridge, United Kingdom).

### Electromyography

Before and after bed rest, wireless surface EMG electrodes (Trigno, Delsys, United States) were placed over M. soleus (SOL), M. gastrocnemius medialis (GM), M. tibialis anterior (TA), M. rectus femoris (RF), M. vastus lateralis (VL) and M. biceps femoris (BF) of the left leg. The longitudinal axes of the electrodes were in line with the presumed direction of the underlying muscle fibers. Interelectrode resistance was reduced by means of shaving and light abrasion of the skin. The EMG signals were wirelessly transmitted to the base station (band-pass filter 20–450 Hz, effective signal gain of 909) and sampled with 2 kHz using the data acquisition unit (Power1401-3, CED, Cambridge, United Kingdom). Surface EMG before and after bed rest only was always performed separately from myotonometry to avoid interference between the two measurement protocols.

### Data Processing

The first two hops as well as hops performed with heel contact were discarded; After removing DC offsets, the EMG signals were rectified. Then, the mean amplitude voltage (MAV) was calculated for every jump for the preactivity phase (50 ms before ground contact touchdown until touchdown). As for force and power, the MAV was then averaged for all valid hops.

### Statistical Analysis

The sample size *n* = 24 participants was adopted according to standardization of bed rest studies proposed by International Space Life Sciences THESEUS Review (Lang et al., 2017). The final sample size was *n* = 23 participants due to one drop-out participant during the BDC period of the RSL study. As no major differences were found between data from MPs on the left and right sides of the body (22 MPs in total), left and right side data were always pooled for analysis (2 × 11 MPs), separated for each group (CTR and JUMP). For statistical analysis IBM SPSS Statistics 23.0 and visualization GraphPad Prism 7.0 (GraphPad Software, Inc., San Diego, CA, United States) were used. The Student’s *t*-test for parametric and Wilcoxon test for non-parametric data analysis of matched pairs with a two tailed *p*-value to analyze changes of variables between time points within groups. Due to low number of cases, the Mann–Whitney-*U*-test was used to compare differences at a time point between groups (JUMP and CTR). Spearman’s correlation coefficient test (Spearman ρ) was applied for closer analysis of variable pairing efficiency. A one-way analysis of variance (ANOVA) (following Kruskal–Wallis test) and Dunn’s multiple comparison test were applied as indicated. Data are given as means ± standard deviation (SD) vs. average baseline (ABL, *x*-axis in graphs) as indicated. Statistical significance was defined as *P* < 0.05, <0.01 or <0.001 if not otherwise indicated.

## Results

### General Findings

**FIGURE 3:**
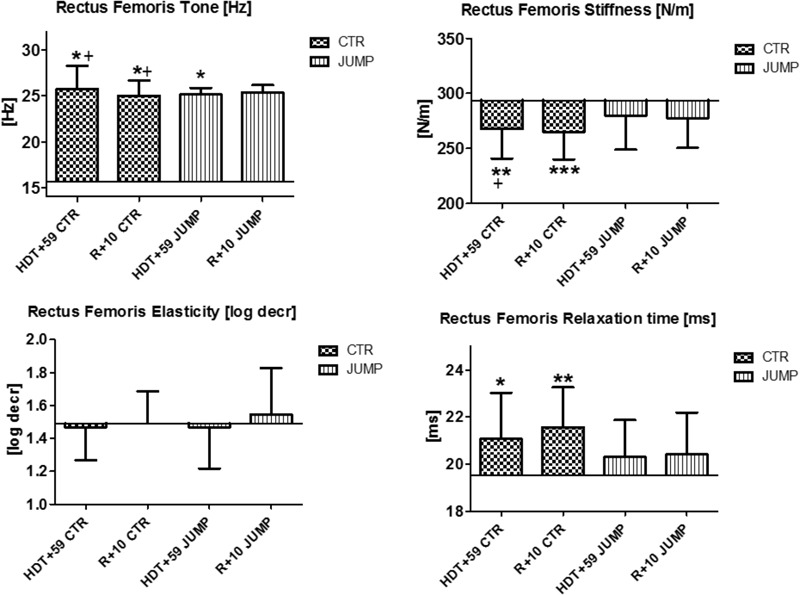
Rectus femoris (MP5/6). Oscillation signal patterns in RSL bed rest study. ABL = Average Baseline (Tone HDT+1 = 15.64; Stiffness HDT+1 = 293.71; Elasticity HDT+1 = 1.49; Relaxation HDT+1 = 19.51). ^∗^*p* < 0.05, ^∗∗^*p* < 0.01, ^∗∗∗^*p* < 0.001 ± SD, +Spearman ρ.

**FIGURE 4:**
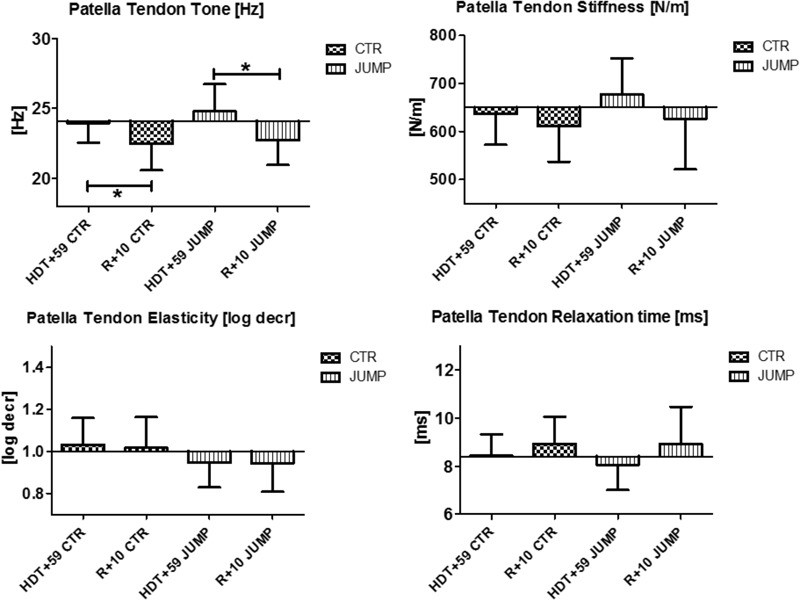
Patellar Tendon (MP7/8). Oscillation signal patterns in RSL bed rest study. ABD, average baseline data (Tone HDT+1 = 24.1; Stiffness HDT+1 = 650.3; Elasticity HDT+1 = 1.0; Relaxation HDT+1 = 8.4). ^∗^*p* < 0.05 ± SD, +Spearman ρ.

**FIGURE 5:**
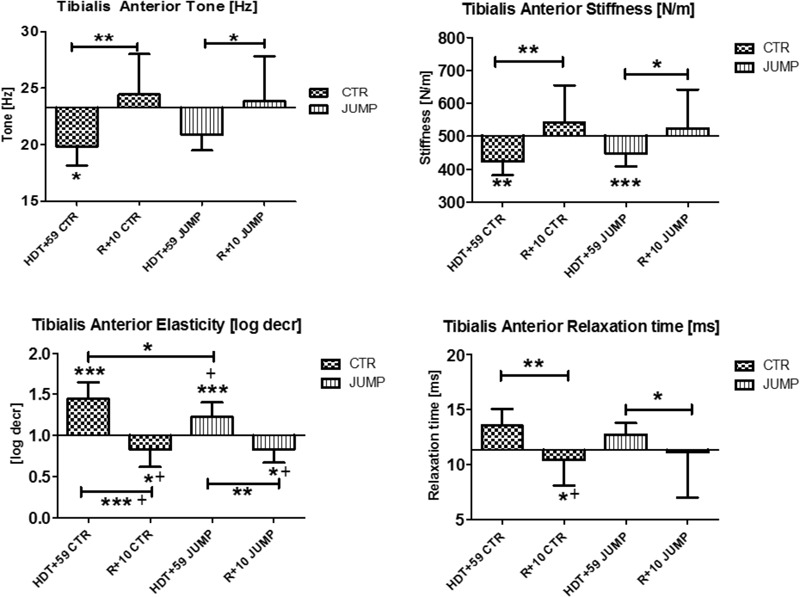
Tibialis anterior (MP9/10). Oscillation signal patterns in RSL bed rest study. *P*-values (asterisks) refer to each graph. Error bars = SD (standard deviation). ABD = average baseline data (Tone HDT+1 = 23.29; Stiffness HDT+1 = 502.43; Elasticity HDT+1 = 1.0; Relaxation HDT+1 = 11.33). ^∗^*p* < 0.05, ^∗∗^*p* < 0.01, ^∗∗∗^*p* < 0.001 ± SD, +Spearman ρ.

**FIGURE 6:**
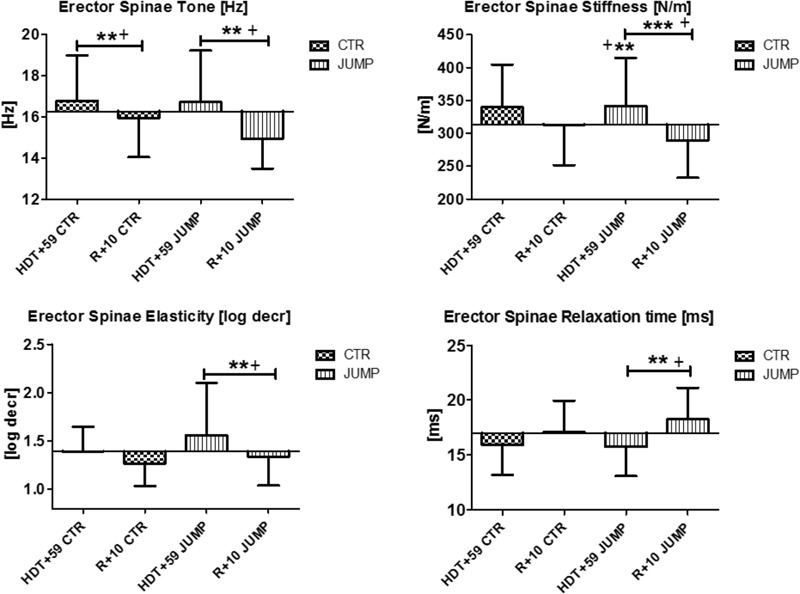
Erector spine (MP11/12). Oscillation signal patterns in RSL bed rest study. *P*-values (asterisks) refer to each graph. Error bars = SD (standard deviation). ABD = Average baseline data (Tone HDT+1 = 16.27; Stiffness HDT+1 = 313.48; Elasticity HDT+1 = 1.39; Relaxation HDT+1 = 17.0). ^∗∗^*p* < 0.01, ^∗∗∗^*p* < 0.001 ± SD, +Spearman ρ.

**FIGURE 7:**
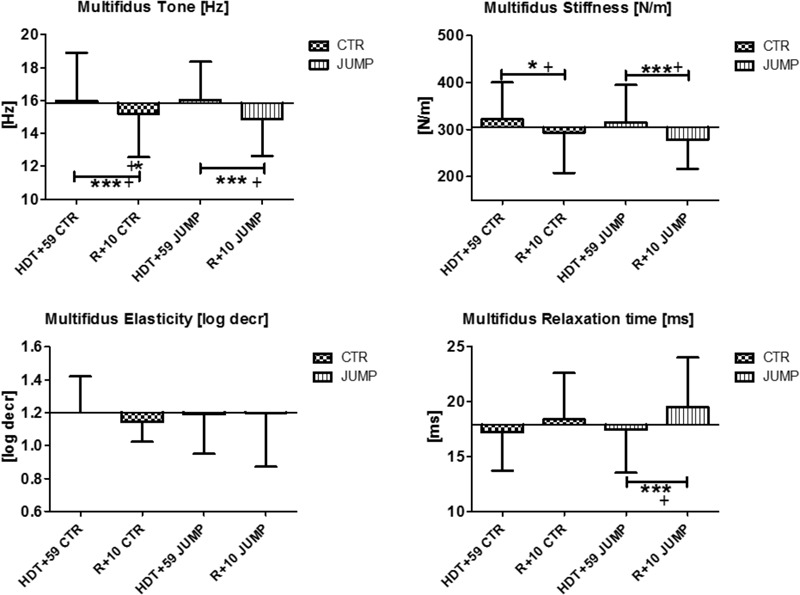
Multifidus (MP13/14). Oscillation signal patterns in RSL bed rest study. ABD = Average baseline data (Tone HDT+1 = 15.85; Stiffness HDT+1 = 304.10; Elasticity HDT+1 = 1.2; Relaxation HDT+1 = 17.9). ^∗^*p* < 0.05, ^∗∗∗^*p* < 0.001 ± SD, +Spearman ρ.

**FIGURE 8:**
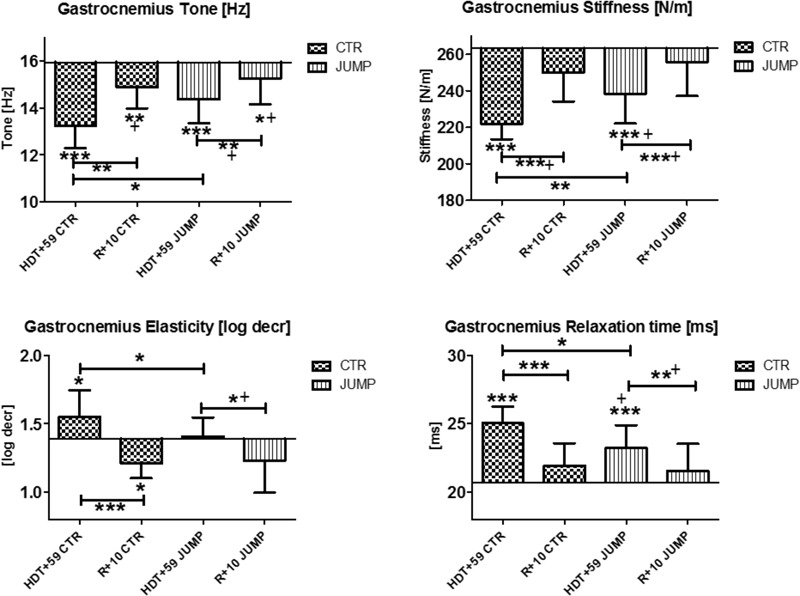
Gastrocnemius, medial head (MP 15/16). Oscillation signal patterns in RSL bed rest study. ABD = average baseline data (Tone HDT+1 = 15.94; Stiffness HDT+1 = 263.22; Elasticity HDT+1 = 1.39; Relaxation HDT+1 = 20.69). ^∗^*p* < 0.05, ^∗∗^*p* < 0.01, ^∗∗∗^*p* < 0.001 ± SD, +Spearman ρ.

**FIGURE 9:**
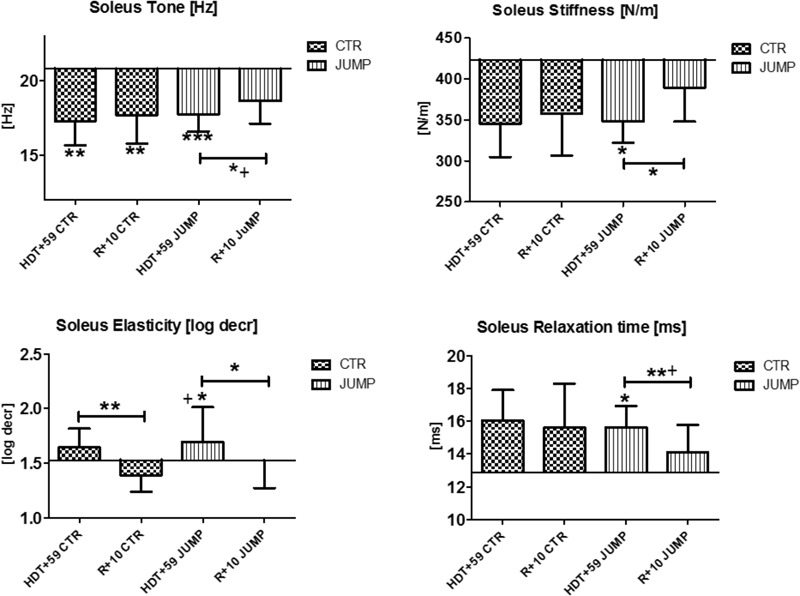
Soleus (MP17/18). Oscillation signal patterns in RSL bed rest study. ABD = average baseline data (Tone HDT+1 = 20.77; Stiffness HDT+1 = 423.33; Elasticity HDT+1 = 1.53; Relaxation HDT+1 = 12.89). ^∗^*p* < 0.05, ^∗∗^*p* < 0.01, ^∗∗∗^*p* < 0.001 ± SD, +Spearman ρ.

**FIGURE 10:**
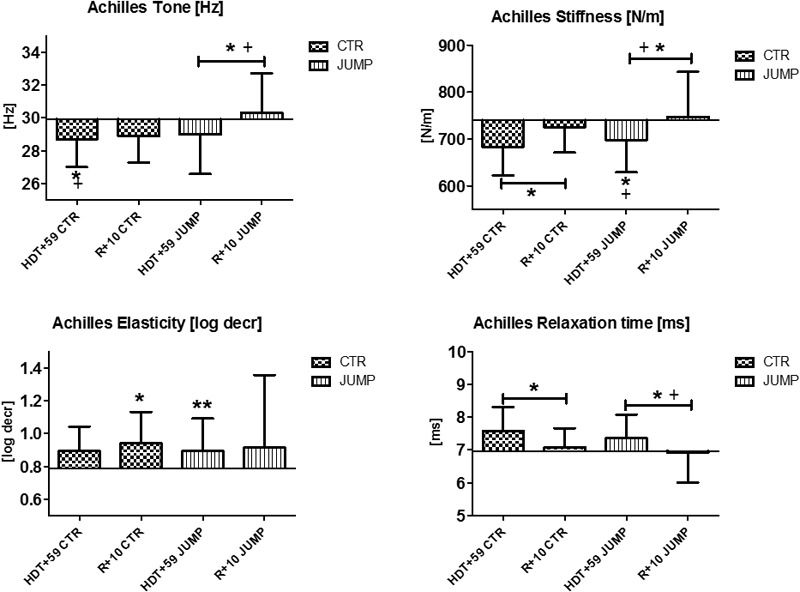
Achilles tendon (MP19/20). Oscillation signal patterns in RSL bed rest study. ABD = Average baseline data (Tone HDT+1 = 29.90; Stiffness HDT+1 = 739.32; Elasticity HDT+1 = 0.79; Relaxation HDT+1 = 6.96). ^∗^*p* < 0.05, ^∗∗^*p* < 0.01 ± SD, +Spearman ρ.

**FIGURE 11:**
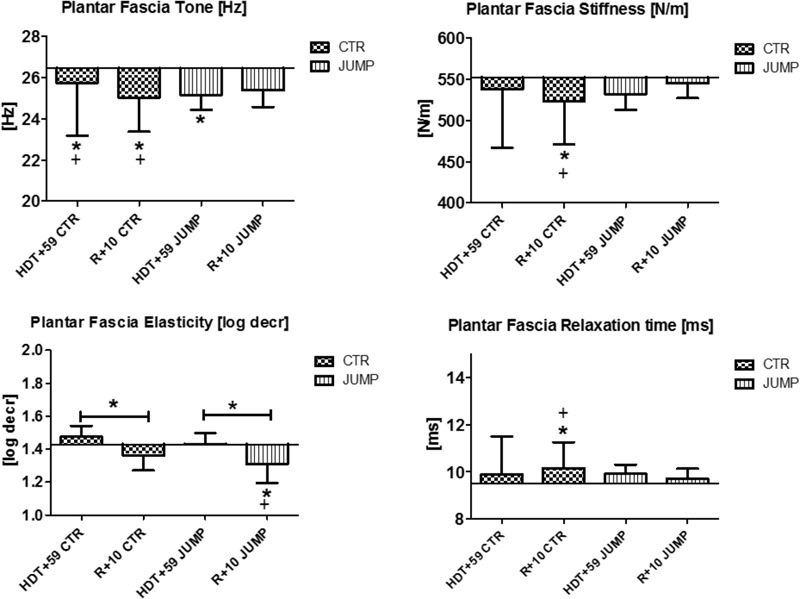
Plantar fascia (MP21/22). Oscillation signal patterns in RSL bed rest study. ABD = Average baseline data (Tone HDT+1 = 26.44; Stiffness HDT+1 = 552.05; Elasticity HDT+1 = 1.43; Relaxation HDT+1 = 9.50). ^∗^*p* < 0.05 ± SD, +Spearman ρ.

**FIGURE 12:**
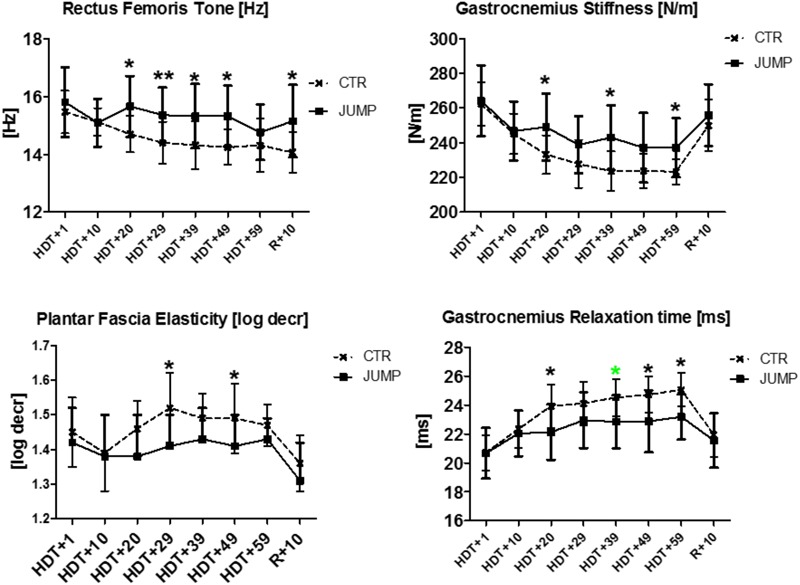
Biomechanical and viscoelastic property changes at different time intervals during RSL bed rest. First changes between groups are detectable in Rectus femoris [tone, Hz], Gastrocnemius (stiffness [N/m], relaxation time [ms]) from HDT+20 onwards (3rd week in bed rest), as well as in Plantar fascia (elasticity, log decrement), from HDT+29 onwards (4th week in bed rest). Mann–Whitney-*U* test, Spearman ρ, ^∗^*p* < 0.05, ^∗∗^*p* < 0.01.

The most striking changes were seen in the muscles of thigh and calf, and as expected, the shoulder muscles showed little change over the bed rest period (**Table 1**). The results from body MPs with robust changes in one or more test parameters only (cf. close circles in **Figure 1**) are presented as bar graphs as data base for definition of bed rest, training and reconditioning effects (**Figures 3–11**). The significant parameter changes notably found in either MPs or between groups (CTR vs. JUMP) at various time point intervals (∼10 days intervals from HDT+10 to HDT+59) during the bed rest period are highlighted in a separate figure (**Figure 12**) as additional reference.

**Table 1:** Neck and Shoulder Muscle biomechanical parameter value changes.

Neck/Shoulder muscle (*n* = 11)		Tone [Hz]	Stiffness [N/m]	Elasticity (log decr)	Relaxation time [ms]
Trapezius MP1,2	CTR Recov JUMP Recov	0.42 ± 1.24 0.32 ± 0.85 0.54 ± 1.37 0.68 ± 0.64^∗^ *p* = 0.0497	6.10 ± 24.56 0.84 ± 16.99 8.08 ± 26.62 10.13 ± 12.84	0.08 ± 0.13^∗^ p = 0.0486 0.04 ± 0.13 0.04 ± 0.19 0.07 ± 0.20	0.96 ± 3.10 0.82 ± 1.92 1.34 ± 2.53 1.50 ± 2.41^∗^ p = 0.0309
Deltoideus MP3,4	CTR Recov JUMP Recov	0.42 ± 1.01 0.54 ± 1.13 0.35 ± 0.89 0.36 ± 0.91	16.10 ± 22.15 14.96 ± 36.06 0.06 ± 25.17 5.68 ± 28.32	0.01 ± 0.08 0.01 ± 0.07 0.02 ± 0.09 0.01 ± 0.09	0.86 ± 1.56 0.70 ± 1.93 0.23 ± 1.42 0.47 ± 1.58

*Biomechanical data collected from neck and shoulder muscle skin measurement points (Trapezius MP1,2 and Deltoideus MP3,4) in bed rest (HDT+59 vs. HDT+1) and recovery (R+10 vs. HDT+59) in both groups (CTR and JUMP). No significant changes in either parameters tested found in supine bed rest. ^∗^Note parameter value changes [Hz, ms] in trapezius (*p* < 0.05) following return to daily life after 10 days (R+10) of reconditioning (recov). Values are parameter value changes ± SD.*

### Shoulder and Neck Muscle

As shown in the body chart (**Figure 1**) the myotonometric protocol started with two upper body muscles from the neck/shoulder region (trapezius and deltoid, M1/2 and MP3/4) with the expectation of only minimal, if any, unloading-induced oscillation signal pattern changes in bed rest vs. ABL. The neck trapezius and shoulder muscle deltoideus were considered as internal controls (neutral reference control, open circles, **Figure 1**) lacking either bed rest or training effects in the experimental set-up of the study in either groups (CTR and JUMP). Following reconditioning (R+10), however, the trapezius in the JUMP group, revealed altered tone [Hz] and relaxation [ms,] parameter changes vs. CTR (**Table 1**).

### Leg Muscles, Tendons and Fascia

In rectus femoris (MP5/6), tone [Hz] was reduced and relaxation time [ms] increased at the end of bed rest in both CTR and JUMP groups, showing bed rest effects but no particular training effect in either group (**Figure 3**). However, muscle stiffness [N/m] was reduced in the CTR group (bed rest effect) but remained unchanged in the JUMP group (positive training effect) at the end of bed rest. During bed rest time intervals, rectus femoris tone [Hz] was mostly increased in the JUMP (positive training effect) vs. CTR group (decreased values reflecting a bed rest effect) at different time points between groups starting from HDT+20 onward (**Figure 12**). In contrast to muscle tone [Hz], only a trend between group and within group was found for stiffness (Δ [N/m] CTR vs. JUMP, HDT+39 *p* = 0.044; CTR HDT+39 = 265.79 ± 24.38; JUMP HDT+39 = 288.0 ± 30.63) elasticity (Δ [log decr CTR vs. JUMP n.s.]) and relaxation time (Δ [ms] CTR vs. JUMP HDT+49 *p* = 0.023; CTR HDT+49 = 21.28 ± 1.04; JUMP HDT+49 = 19.88 ± 1.64) during bed rest. Following reconditioning, muscle tone [Hz] values were closer to baseline in the JUMP than in the CTR group (reconditioning effect). In rectus femoris (**Figure 3**), key myotonometric parameter changes were found indicating a bed rest effect in both groups: CTR (Δ Hz, N/m and ms) and JUMP (Δ Hz).

In the patellar tendon (MP7/8), altered oscillation signal patterns were not found between groups at the end of bed rest (**Figure 4**). During bed rest time intervals, only a trend for reduced stiffness [N/m], but increased elasticity [log decr] after 4 weeks in bed rest (HDT+29 and HDT+49) was found between and within group. Following reconditioning, however, tendon tone reduction was found in both groups. In the patellar tendon, however, a key parameter change from baseline (Δ Hz) was identified indicating reconditioning effects (**Figure 4**).

In tibialis anterior (MP9/10), changes were found in all four parameters tested at the end of bed rest (i.e., decreased tone/stiffness, increased elasticity/relaxation time values) vs. ABL suggesting strong bed rest effects occurring in both groups (**Figure 5**). In addition, a training effect was also seen by two altered mechanical characteristics reflecting elasticity [log decr] and relaxation time [ms] values in JUMP vs. CTR (**Figure 5**). Following reconditioning all parameters recovered (positive reconditioning effect) in all groups suggesting that a group-dependent reconditioning effect could not be determined by myometry (**Figure 5**). During bed rest time intervals, some between-group changes were seen by one or the other parameter, for example, at mid-bed rest (Δ [Hz] CTR vs. JUMP, HDT+20 = 1.7 *p* = 0.027; HDT+29 = 1.2 *p* = 0.027; Δ [N/m] HDT+20 = 32.5 *p* = 0.043; Δ HDT+29 = 23.4 *p* = 0.037; Δ [ms] HDT+20 = 1.4 *p* = 0.032; Δ HDT+29 = 1.1 *p* = 0.025), or later (Δ [Hz] CTR vs. JUMP, HDT+49 = 1.4 *p* = 0.037; [log decr] HDT+49 = 0.2 *p* = 0.044) in bed rest suggesting oscillation signal pattern changes (higher in tone /stiffness or lower in elasticity and relax. time) detectable in CTR vs. JUMP during bed rest. In tibialis anterior, key parameter changes indicating a bed rest effect were identified in both CTR (Δ Hz, N/m, log decr, ms) and JUMP (Δ N/m, log decr., ms), only one from JUMP (Δ log decr) indicating a training effect, whereas the changes in all other parameters (Δ all four) indicated a reconditioning effect in both CTR and JUMP groups (**Figure 5**).

### Paraspinal Back Muscles

In the erector spine (MP11/12), increased muscle tone [Hz] was detectable at the end of bed rest in both groups (**Figure 6**) suggesting a bed rest effect. However, different stiffness and elasticity values were found in the JUMP group only at the end of bed rest suggesting a strong bed rest effect (increased muscle stiffness) but also a training effect (decreased muscle elasticity) particularly in the JUMP group (**Figure 6**). During bed rest time intervals, a trend was seen particularly in altered erector spinae elasticity values determined in bed rest [e.g., log decr CTR HDT+10 = 1.29 ± 0.19; HDT+20 = 1.32 ± 0.2; HDT+29 = 1.36 ± 0.25; HDT+39 = 1.38 ± 0.21; HDT+49 = 1.37 ± 0.26; JUMP HDT+10 = 1.57 ± 0.66; HDT+20 = 1.51 ± 0.48; HDT+29 = 1.59 ± 0.52; HDT+39 = 1.49 ± 0.43; HDT+49 = 1.40 ± 0.34] whereas all other parameters remained largely unchanged during the mid-course of bed rest (not shown). Following reconditioning, erector spine stiffness and elasticity remained unchanged in CTR (negative reconditioning effect) and were different in the JUMP group (positive reconditioning effect) while tone and relaxation time recovered (positive reconditioning effect) to baseline in the CTR group (**Figure 6**). In erector spinae, one key parameter change indicating a bed rest effect was identified (Δ N/m) in the JUMP group (increased stiffness), none of the parameters indicating training effects, whereas all others indicating reconditioning effects in the CTR (Δ Hz) and JUMP groups (Δ all four).

In the lumbar multifidus (MP13/14), biomechanical properties [Hz, log decr, ms] remained unchanged but stiffness [N/m] was increased in both groups at the end of bed rest suggesting absence of a detectable bed rest effect other than stiffness for this paraspinal back muscle (**Figure 7**). During bed rest time intervals, no major changes for multifidus was found in any parameters or between groups (data not shown). Following reconditioning, oscillation signal patterns were mostly changed (reduced) independently from groups (CTR, JUMP) particularly seen by reduced muscle tone [Hz]. Notably, relaxation time [ms] was, however, different in R+10 JUMP vs. HDT+59 JUMP (**Figure 7**). In multifidus, two altered key parameters were identified in the CTR group (Δ Hz, N/m), and three key parameters (Δ Hz, N/m, ms) in the JUMP group indicating an apparently negative outcome in post- bed rest reconditioning.

### Calf Muscle and Achilles Tendon

The gastrocnemius (medial head, MP15/16) and soleus (MP17/18) of the calf triceps surae showed significant changes of their oscillation signal patterns in bed rest in both the CTR and JUMP groups (**Figures 8, 9**). At the end of bed rest, all four oscillation signal patterns were altered (tone and stiffness reduced, elasticity and relax. time increased vs. ABL) in the medial gastrocnemius, more markedly in the CTR than in the JUMP group, suggesting robust bed rest effects in both groups but also training effects in the JUMP group compared with the CTR group (**Figure 8**). During bed rest time intervals (**Figure 12**), group-related oscillation signal pattern changes were detectable at some time points starting from HDT+20 onward, for example, tone [Hz] (HDT+20 and +59), stiffness (HDT+20, +39, and +59) and relaxation time (HDT+20, +49, and +59). Nevertheless, following reconditioning, all four parameter values recovered within a period of only 10 days (reconditioning effect in both groups). In gastrocnemius, key parameters indicating bed rest effects were identified in the CTR group (Δ all four) but only three (Δ Hz, N/m, ms) in the JUMP group. However, all four key parameters in the JUMP group were different from the CTR group at the end of bed rest thus indicating a positive training effect in bed rest. In addition, changes to all four parameters (Δ all four) were identified in both CTR and JUMP groups indicating a post-bed rest reconditioning effect in both groups.

In the mostly slow type soleus (deep postural part of the calf triceps) a group independent bed rest effect was found in all four parameters [Hz, N/m, Log decr., ms] tested in the study (**Figure 9**) suggesting a bed rest effect in both groups thus lacking a robust training effect in the JUMP group at the end of bed rest (**Figure 9**). Following reconditioning, tone, stiffness and relaxation time, however, recovered faster in the JUMP vs. CTR group (positive reconditioning effect) while elasticity value changes suggested faster recovery effects in the CTR than in the JUMP group (**Figure 9**). During the bed rest time intervals, only a trend was found in the soleus viscoelastic response between groups at any of the time points (data not shown). In soleus, only one key parameter change (Δ Hz) was identified in the CTR group and all four parameters (Δ all four) in the JUMP group at the end of bed rest indicating a negative bed rest effects in the CTR group not seen in the JUMP group.

The Achilles tendon, insertion of the distal calf triceps to the calcaneus, showed variable changes in viscoelastic properties at the end of bed rest, such as reduced tone [Hz] and reduced stiffness [N/m] in both CTR and JUMP groups, and increased numerical values reflecting less elasticity [log decr] in the JUMP group only (**Figure 10**). Following reconditioning, the JUMP group revealed a robust biomechanical property change response by increased tension/tone [Hz] and stiffness [N/m] vs. CTR group that possibly reflect two detectable key mechanical characteristics of Achilles tendon readaptation (positive reconditioning effect) following reloading after bed rest. During bed rest time intervals, parameter value curves almost matched between groups (data not shown). In Achilles tendon, one key parameter change was identified in the CTR group (Δ Hz) indicating a bed rest effect, and two others in the JUMP group (Hz, log decr) indicating lack of bed rest effects similar to the CTR group at the end of bed rest. No change in any key parameter was found in the JUMP group indicating a training effect at the end of bed rest. However, two key parameter changes in the CTR group (Δ N/m, log decr.) and three others in the JUMP group (Δ Hz, N/m, ms) were identified at R+10 vs. HDT+59 suggesting differential group-related reconditioning effects.

### Plantar Fascia

The plantar fascia supported by the flexor digitorum brevis (MP21/22) on the sole of the foot with potential sensitivity to body weight unloading and reloading following extended disuse was finally measured at the end of the session in each group (**Figure 11**). In the sole of the foot myofascial structures, reduced tone [Hz] was determined at the end of bed rest in both CTR and JUMP groups suggesting presence of a robust bed rest effect but there was no detectable training effect in the JUMP group (**Figure 11**). Following reconditioning, tone [Hz], stiffness [N/m] and elasticity [Log decr] values were quite different in CTR vs. JUMP groups suggesting these mechanical characteristics as evident key parameters reflecting differential reconditioning effects (**Figure 11**). During bed rest, however, only a trend was found, for example, by increased elasticity [log decr] values in CTR vs. JUMP groups at two mid-bed rest time points (HDT29 and HDT49) suggesting altered viscoelastic property changes related to decreased elasticity in the plantar fascia/*flexor digitorum brevis* myofascial structures following unloading conditions in bed rest (**Figure 12**). At the plantar fascia MP, tone was the key parameter (Δ Hz) that was always changed vs. baseline in both CTR and JUMP groups, suggesting a strong bed rest effect present in both groups that could not be mitigated for example by reactive jumps in bed rest (i.e., no key parameter was identified indicating a training effect in JUMP). Nevertheless, one key parameter change (Δ log decr) was documented after the bed rest period suggesting a reconditioning effect shown by the different tissue elasticity parameter found in both groups.

### Muscle Physiology

Results from three different time points (BDC-1, R+0, and R+7) show only trends in either groups before and after bed rest but with variable individual muscle outcome (**Table 2**).

**Table 2:** Muscle physiology data from RSL study groups before and after bed rest.

Muscle/Time points		CTR (*SD*)	JUMP (*SD*)	*P*-values^∗^
SOL	BDC-1 R+0 R+7	0.09 (0.02) 0.09 (0.04) 0.07 (0.03)	0.07 (0.03) 0.07 (0.02) 0.07 (0.03)	0.70
GM	BDC-1 R+0 R+7	0.15 (0.06) 0.12 (0.04) 0.12 (0.03)	0.13 (0.08) 0.13 (0.07) 0.13 (0.03)	0.11
TA	BDC-1 R+0 R+7	0.02 (0.01) 0.02 (0.00) 0.02 (0.01)	0.03 (0.01) 0.02 (0.02) 0.02 (0.01)	0.10
VL	BDC-1 R+0 R+7	0.11 (0.09) 0.07 (0.05) 0.06 (0.03)	0.08 (0.05) 0.10 (0.07) 0.11 (0.07)	0.07
RF	BDC-1 R+0 R+7	0.05 (0.05) 0.05 (0.03) 0.04 (0.03)	0.04 (0.02) 0.04 (0.02) 0.04 (0.02)	0.28

*Muscle performance and EMG. Means and standard deviations (SD) for all participants of the control group (CTR, *n* = 10) and the training group (JUMP, n = 11) for three time points (BDC-1, R+0, and R+7). ^∗^*P*-values refer to the results of the repeated measures ANOVA for the time points directly before and after bed rest (BDC-1 and R+0). SOL, soleus; GM, medial gastrocnemius; TA, tibialis anterior; VL, vastus lateralis; RF, rectus femoris.*

### Intramuscular Collagen-1 Network (Soleus Biopsy)

**FIGURE 13:**
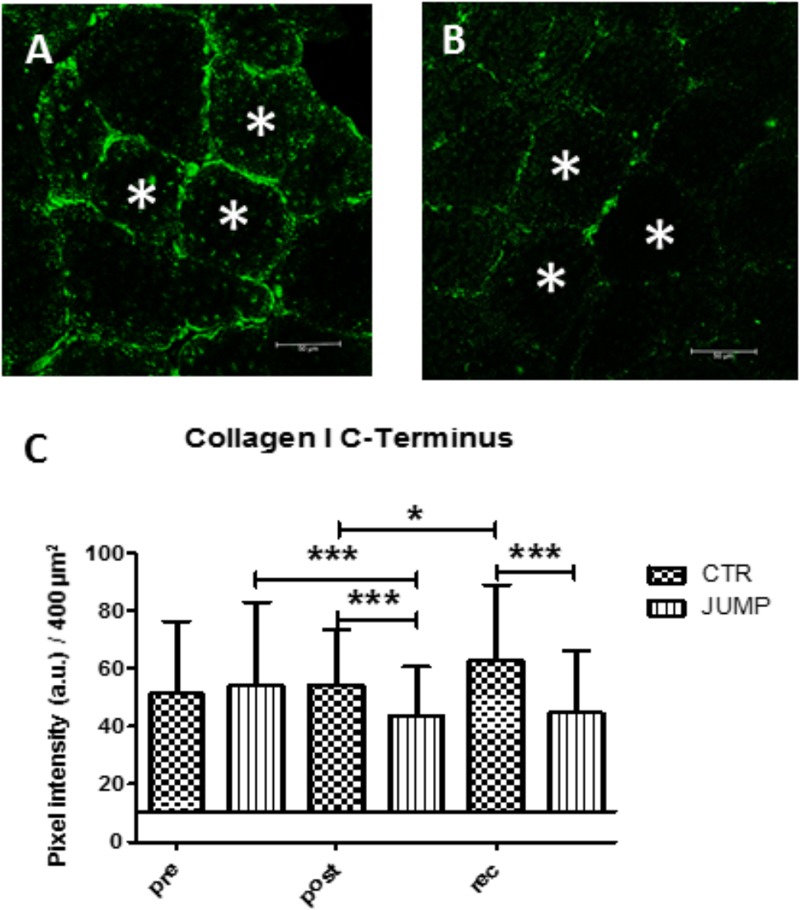
Immunohistochemistry of collagen-1 network adaptation in RSL soleus biopsy. **(A)**, CTR group (post-BR HDT+59) shows increased collagen-1 immunoreactivity at peri- and endomysial tissue around cross-section myofibers, **(B)**, JUMP group (post-BR HDT+59) shows reduced collagen-1 immunoreactivity, **(C)** Quantification by area pixel-intensity determination (200 × 200 μm^2^) in each group at bed rest start (Pre), end (Post) and recovery (rec). ^∗^*p* < 0.05, ^∗∗∗^*p* < 0.001 (*n* = 100 myofibers/cryosection). a.u., arbitrary units, Bar = 50 μm.

Results show that the dense collagen network detectable in the peri- / and endomysial connective tissue spaces ensheathing muscle fibers typically seen in normal soleus biopsies before the start of bed rest (CTR and JUMP) showed reduced immunostaining patterns (**Figures 13A,B**). However, these changes only occurred in the JUMP group at the end of bed rest, suggesting exercise-induced remodeling of the collagen fiber network in muscle following the RSL training protocol in bed rest. Quantification of results (**Figure 13C**) confirmed reduced density patterns of the immunostained collagen-1 network in JUMP vs. CTR groups at the end of bed rest that persisted also at 10 days of recovery from the bed rest period (**Figure 13C**).

## Discussion

### Major Findings and Study Outcome

In the present study a set of dynamic oscillation mechanosignals (Hz, N/m, log decr, ms) collected by a non-invasive digital palpation device (MyotonPRO) was used to identify key determinants of biomechanical parameter and viscoelastic property changes in global and postural human skeletal muscle, tendon and fascia in a bed rest study. Recordings were obtained from a bed rest only group (CTR) and a bed rest exercise group (JUMP) performing reactive jumps as a countermeasure, at the start of bed rest, during, and 10 days after bed rest (60 days RSL study 2015–2016).

A primary study outcome was to provide an accurate and easy operation protocol for objective monitoring of biomechanical and viscoelastic property changes in surface muscle and myofascial structures of the HRMT following chronic disuse in bed rest without and with exercise. A second study outcome was the identification of major key baseline oscillation signal patterns obtained from each of the functionally important components of the HRMT in disuse and exercise in otherwise healthy bed rest participants for assessment of general muscle health. A further study milestone was to achieve some correlation between biomechanical property changes found in muscle, tendon and fascia that shape the HRMT with functional data from RSL study performance tests (one leg hops, surface EMG), for example, from the leg jumping-relevant functional muscle chain in both CTR and JUMP study groups. Digital palpation of surface body muscle and associated myofascial components in bed rest allowed us to monitor the outcome of (i) the negative effects of bed rest (bed rest effect) on a set of functionally important components of the HRMT system (i.e., changes in muscle tone, stiffness, elasticity, relaxation time, tendon and fascia stiffness), (ii) the training effects seen during bed rest (i.e., preservation of biomechanical and viscoelastic properties in functional muscle chains in JUMP vs. CTR groups) induced by a high load exercise protocol (reactive jumps as countermeasure in bed rest), and (iii) the reconditioning effects (e.g., baseline recovery of calf muscle, dorsiflexors, tendons and fascia in JUMP vs. CTR) seen after 10 days of recovery from bed rest. Reliability statistics and reproducibility of data sets obtained by the MyotonPRO technology was shown in previous multidisciplinary studies on some of the muscle, tendon and fascia relevant for the present study (Zinder and Padua, 2011; Agyapong-Badu et al., 2013; Fröhlich-Zwahlen et al., 2014; Pruyn et al., 2014; Schneider et al., 2015; Orner et al., 2017). During the measurement sessions in the present bed rest study two independent operators thus allowed for reproducible data collection from some of the functional components that shape the HRMT.

### Tone and Stiffness of Skeletal Muscle and Tendons in Disuse

The most striking changes were seen in the muscles of thigh/rectus femoris) and calf (gastrocnemius, soleus), as well as in Achilles tendon and plantar fascia with myotonometry used during bed rest. Another method to document biomechanical properties in stiffness and plasticity of disused tendons and myotendinous junctions was used previously in the knee extensors (rectus femoris of the Quadriceps) and plantar flexors (Triceps surae) following 20 days of bed rest (Kawakami et al., 2000; Lapole and Pérot, 2011) as well as adaptation and maladaptation following muscle disuse/unloading and resistive exercise thus underlining the viscoelastic function of the muscle-tendon-bone interface in muscle physiology and its obvious maladaptation during extended disuse in bed rest. Data from a short-term (3–5 days) “dry water immersion” study noted a decrease in muscle stiffness of the calf triceps surae (Koryak, 2014) and rectus femoris (Demangel et al., 2017; De Abreu et al., 2017), whilst earlier work proposed that muscle stiffness was decreased throughout global muscle groups during real microgravity (Gallasch and Kozlovskaya, 1998; Lambertz et al., 2001; Lambertz et al., 2003). As expected, the shoulder muscles showed little change over the bed rest period. However, we did find changes in both neck, shoulder and even back muscle biomechanical properties after 10 days of recovery from bed rest. This is possibly induced by the known variability in every day shoulder muscle movements (Liu et al., 2017) and the reloading forces effect in the neck/shoulder due to anti-orthostatic vs. orthostatic (from supine to upright) body position changes in bed rest not considered as part of the present study aims.

### Negative Bed Rest Effects on the HRMT System

The lower body musculoskeletal tissues, most strikingly in the calf, showed changes in biomechanical and viscoelastic properties that reflect known disuse-induced atrophy changes in a characteristic global vs. postural pattern (Pavy-Le Traon et al., 2007). The present biomechanical property changes in CTR are also reflected by significant decrease in functional muscle data (isometric MVC) recorded from the same RSL study participants (Kramer et al., 2017a) at bed rest start (BDC-1) and end (R+0) suggesting strong data consistency through correlations between biomechanical and functional muscle data collected from the bed rest participants. In particular, major key parameters of disuse were found in the CTR group such as muscle tone [Hz], stiffness [N/m], and elasticity (log decrement) mostly detected from the back muscles (erector spinae, multifidus), but also in the legs, in the patellar and Achilles tendons, gastrocnemius, soleus and tibialis anterior muscles, commonly known to have a role in functional control of human body stabilization in standing, walking and other exercises in everyday life, and for example, also observed after gravitational unloading (Rukavishnikov et al., 2017). Similar changes to general skeletal muscle tone and stiffness, which supposedly develops consistently over time in the almost relaxed musculature of the partially unloaded human body in a supine posture, were previously studied in the lumbar multifidus muscles of women during 120 days of bed rest confinement (Holt et al., 2016), lower extremity calf triceps muscle and myotendinous stiffness (Koryak, 2014) using, however, conventional electrically evoked neurophysiological methods (maximally voluntary contraction, isometric twitch and tetanic contractions, EMG). Myotonometry is able to reliably measure tone and stiffness in erector spinae in normal healthy subjects (Lohr et al., 2018). At present it remains to be shown if changes in tone and stiffness of erector spinae (and its muscular subgroups such as longissimus dorsi and multifidus) in bed rest may be due to uncontrolled movements of the trunk (e.g., short trunk and head lifting and lateral uncontrolled movements during sleeping) or subject to unloading effects in bed rest disuse.

Non-invasive myotonometry was able to document more acute negative effects on resting muscle tone in the 3 days dry immersion (3DI)-unloaded rectus femoris (Demangel et al., 2017), and as shown for muscle, tendon and fascia of the HRMT in the present long-term bed rest study.

Immunohistochemistry in biopsy sections from the disused CTR calf soleus from the present long-term bed rest study showed increased intramuscular collagen-1 network immunoreactivity indicative of abnormal cellular adaptation to reduced mechanical stimulation (reduced muscle contraction forces), possibly impaired by stiffer intramuscular peri- and endomysial layers around muscle fiber bundles. From the non-muscular HRMT components investigated here, patellar tendon, Achilles tendon and plantar fascia viscoelastic properties were considerably changed in chronic disuse, suggesting profound functional tissue alterations induced by chronic disuse in bed rest in the absence of normal mechanical loading conditions such as standing, walking or exercise with some clinical relevance in otherwise healthy but sedentary populations (Sohirad et al., 2017).

### Training Effects of Reactive Jumps on HRMT During Bed Rest

Myotonometric determination of biomechanical parameters indicating individual muscle stiffness, for example of the hip rectus femoris and calf gastrocnemius, generated reliable physical performance markers (sprinting, agility, jumping) reflected by differential lower body stiffness in Australian footballers to better monitor strength and conditioning in athlete training (Pruyn et al., 2014). In the present study we provide evidence for training effects as reflected by key myotonometric characteristics of functional muscle groups and associated myofascial components affected by a highly demanding countermeasure protocol (reactive jumps on sledge system) during bed rest. The sledge jump system has been previously successfully applied in performance studies (Kramer et al., 2010, 2012). In the JUMP group some of the biomechanical and viscoelastic parameter values (tone, stiffness, elasticity) increased or decreased (i.e., erector spinae, soleus, gastrocnemius, tibialis anterior), tendon (Achilles tendon) and fascia (plantar fascia), while others remained unchanged at the end of bed rest vs. the start, such as tone and stiffness in rectus femoris, all four parameters in gastrocnemius, as indicators for a training effect elicited by the countermeasure protocol during bed rest. The present biomechanical property changes in JUMP, for example, well correlated with altered isometric maximally voluntary contraction force (MVC) recordings determined at bed rest start (BDC-1) and end (R+0) in at least knee extensors and plantarflexors of the same bed rest participants (Kramer et al., 2017a) confirming a strong correlation between biomechanical and functional data from RSL bed rest participants. At the time course intervals (approximately every 10 days) during bed rest, mainly the leg muscles and plantar fascia, but not Achilles tendon, showed between-group difference in changes in some biomechanical parameter values. These differences indicated key determinants helpful to monitor gradual changes in disuse responses in a given muscle or related fascia, as for example reflected by tone / tension changes [Hz] in rectus femoris and gastrocnemius, stiffness [N/m] in tibialis anterior and elasticity changes [log decr.] in gastrocnemius, elasticity [log decr] changes in plantar fascia, mostly found at the time intervals from the second half of the bed rest period (i.e., HDT-29) onward (cf. **Figure 12**).

Positive correlations with some but not all four biomechanical parameters measured were mainly found in lumbar back and leg muscles, and the myofascial functional chain, likely due to the direct targeting of the reactive jumping training protocol prescribed in this study. Perhaps reactive jumps also provided, at least for very short time periods of contacts to the foot plates, additional plantar mechanical support to activated leg muscle tension as previously suggested from short duration dry immersion studies (Miller et al., 2004; Ogneva et al., 2011). Immunohistochemistry in RSL SOL biopsy tissue sections from the JUMP group revealed a moderately dense intramuscular collagen-1 network vs. CTR. As discussed before such findings may well reflect disproportionate connective tissue remodeling as part of mechanically induced cellular and molecular mechanisms in muscle tissue probably also occurring in the other myofascial components during HRMT adaptation otherwise preserved by exercise as a countermeasure.

### Reconditioning Effects on HRMT Between Groups After Bed Rest

When bed rest participants returned back to normal life (i.e., upright body position, normal daily activities) from their strictly confined anti-orthostatic supine/prone bed rest period of 60 days, some mechanical and viscoelastic numerical parameter values showed differences between R+10 (10 days of reconditioning) vs. HDT+59 (bed rest end) which is a likely indicator for positive or even negative reconditioning effects seen in muscle, tendon and fascia of the HRMT system within the second week of post-bed rest recovery. From immunostaining experiments, which were also part of the present study, a moderate density of the intramuscular anti-collagen-1 density network in the soleus biopsy sections seen in the JUMP group at the end of bed rest persisted within 10 days after bed rest confinement as a cellular and tissue preservation sign, either in terms of a lasting (after-effect) or reloading effect as re-adaptation to normal daily activities in the upright body position seen after just 10 days of reconditioning.

### Myotonometric Data Translated to Muscle Cell Physiology and Function

The question arises how biomechanical and viscoelastic property changes in biological soft tissue can be translated to meaningful concepts about the underlying cellular and molecular mechanisms of the functional components that shape the HRMT in normal life or in other environmental challenging conditions. Apart from the noteworthy exercise-induced preservation of the intramuscular collagen network density in postural soleus in bed rest shown here and suggested previously for the myofascial tissue (Schleip et al., 2006), work in the literature offers several molecular structural elements as candidates generating the resting muscle tone. Possible elements include intrinsic forces of acto-myosin cross-bridging (Campbell and Lakie, 1998), the non-crossbridge stiffness and spring-like sarcomeric giant proteins titin (Labeit et al., 2003; Colombini et al., 2016) and nebulin (Horowits et al., 1986), contractile myofibroblast cells (i.e., α-smooth muscle actin immunopositive stress-fiber bundles) found in superficial body fascia (Staubesand and Li, 1996). Further possible candidates are the viscoelastic properties of the extracellular matrix including proteoglycans and glycosaminoglycans, collagen fiber cross-links, and non-collagenous link proteins (Proske and Morgan, 1999). In fact, also metabolic activity should be considered as a possible attribute to resting-muscle mechanical work (McKay et al., 2010). In disuse, desmin and actinin levels were found to be altered in human skeletal myofibers as possible biomarkers of altered muscle tone and stiffness (Ogneva et al., 2011). For a better understanding of the complex molecular and cellular mechanisms, further investigations in various conditions of HRMT adaptation are needed.

In contrast to previous studies with young adults and athletes in sport (Gavronski et al., 2007; Nair et al., 2016) the present biomechanical and viscoelastic property changes did not fully overlap with the outcome of muscle performance tests (i.e., one leg hops synchronized to EMG signals) performed on the study participants before and after bed rest. The functional tests rather showed individual trends, for example, in the thigh rectus femoris and vastus lateralis, in calf gastrocnemius (plantar flexor), and in the co-contraction dorsiflexor muscle tibialis anterior (**Table 2**). This may be explained by the fact that, at least in bed rest spatial and temporal changes in biomechanical and viscoelastic soft tissue parameters of skeletal muscle tissue (EMG silent) may not occur in parallel to functional neuromuscular changes initiated by the jump exercises detectable by surface EMG signal recordings. Further investigations to elucidate the observed mismatch between biomechanical and functional data in disuse are needed for example to better understand the mechanisms of lateral force transmission perturbations between muscle fibers and associated myofascial tissue and neuromuscular adaptation mechanisms under conditions of extended disuse and exercise.

### Study Limitations

Surface EMG recordings to check for completeness of resting muscle tone status (McKay et al., 2010) prior to myotonometry sessions in bed rest were omitted due to study protocol-related constraints (e.g., implementation of >1.500 single pre-, post- and recovery measurements to study schedule, complexity of experimental set-up, risk of local skin reactions, stress factors).

## Conclusion

The present findings provide further insights to the structural and functional responses of the HRMT system to chronic disuse in bed rest. With the help of the non-invasive myotonometric protocol we were able to define robust bed rest effects in the CTR (without exercise prescriptions) as well as significant training effects in the exercise group (reactive jumps) that we found in global as well as postural muscle groups including tendon and fascia in otherwise healthy male bed rest participants. Soft tissue region and tissue specific biomechanical characteristics found in global and local muscle and myofascial tissue at resting conditions appear to precede functional changes (i.e., property changes prior to functional changes). This allows for real time monitoring of the deconditioned HRMT system in bed rest and provides an early assessment tool if routine (and often highly demanding) neuromuscular tests may not be available, for example, in open field study settings. Myotonometric testing in disuse and exercise proved to be a less costly, easier and more rapid and resilient method than conventional methods and it may be used as a practical evidence-based outcome evaluation tool for an efficient physical countermeasure outcome on the human muscle status/condition in prolonged periods of disuse, perhaps even replacing strenuous functional testing in future human analog studies. The present findings may be even more helpful for a better treatment efficacy in various healthy and clinical populations but also for the monitoring of muscle health status of crew during pre and inflight mission training, and reconditioning thereafter in personalized space medicine.

## Author Contributions

DB, MS, and MJS: Conceptualization. VZ, SD, AK, and BS: Data curation. BS and DB: Formal analysis. DB: Funding acquisition. DB, BS, MS, and AK: Investigation. AK, DB, VZ, SD, and AV: Methodology. EM, UL, and DB: Project administration. AP: Technical Support. EM and UL: Resources and staff from DLR:envihab human space life science facility, Cologne. UL, RV: Supervision. BS, MS, and DB: Validation. DB, BS, and MJS: Writing – original draft. All authors: Writing – review and editing.

## Conflict of Interest Statement

AP was employed by Company Myoton AS, Estonia. The remaining authors declare that the research was conducted in the absence of any commercial or financial relationships that could be construed as a potential conflict of interest.

## Footnotes

1 http://www.dlr.de/envihab/

2 Leica-microsystems.com

## Abbreviations

ABL: average baseline
BDC: baseline data collection
BMI: Body Mass Index
CTR: control group
cm: centimeter
D: logarithmic decrement [no unit]
DLR: Deutsches Zentrum für Luft- und Raumfahrt
e.g.: example given
ESA: European Space Agency
F: [Hz] Natural oscillation frequency
GM: Gastrocnemius (medial head)
HDT: Head-down tilt
HRMT: Human Resting Muscle Tone
JUMP: reactive jump group (exercise group)
L: Lumbar level
MMG: Mechanomyography
MPs: Measure points (skin marks)
MVC: Maximal voluntary contraction
N: Newton
R: Relaxation time [ms]
R+: Reconditioning (recovery) plus
RF: Rectus femoris (mid-head of quadriceps femoris)
RSL: reactive jumps on sledge (system)
S: Stiffness [n/m]
sEMG: Surface electromyography
SOL: Soleus muscle
SWE: Shear wave elastography
TA: Tibialis anterior
Th: Thoracic level
VL: Vastus lateralis (lateral head of quadriceps femoris)
